# Teenagers and Texting: Use of a Youth Ecological Momentary Assessment System in Trajectory Health Research With Latina Adolescents

**DOI:** 10.2196/mhealth.2576

**Published:** 2014-01-24

**Authors:** Carolyn Garcia, Rachel R Hardeman, Gyu Kwon, Elizabeth Lando-King, Lei Zhang, Therese Genis, Sonya S Brady, Elizabeth Kinder

**Affiliations:** ^1^University of MinnesotaSchool of NursingMinneapolis, MNUnited States; ^2^University of MinnesotaSchool of Public HealthMinneapolis, MNUnited States; ^3^University of MinnesotaMinneapolis, MNUnited States

**Keywords:** texting, data collection, intervention research, longitudinal, trajectory

## Abstract

**Background:**

Adolescent females send and receive more text messages than any others, with an average of 4050 texts a month. Despite this technological inroad among adolescents, few researchers are utilizing text messaging technology to collect real time, contextualized data. Temporal variables (ie, mood) collected regularly over a period of time could yield useful insights, particularly for evaluating health intervention outcomes. Use of text messaging technology has multiple benefits, including capacity of researchers to immediately act in response to texted information.

**Objective:**

The objective of our study was to custom build a short messaging service (SMS) or text messaging assessment delivery system for use with adolescents. The Youth Ecological Momentary Assessment System (YEMAS) was developed to collect automated texted reports of daily activities, behaviors, and attitudes among adolescents, and to examine the feasibility of YEMAS. This system was created to collect and transfer real time data about individual- and social-level factors that influence physical, mental, emotional, and social well-being.

**Methods:**

YEMAS is a custom designed system that interfaces with a cloud-based communication system to automate scheduled delivery of survey questions via text messaging; we designed this university-based system to meet data security and management standards. This was a two-phase study that included development of YEMAS and a feasibility pilot with Latino adolescent females. Relative homogeneity of participants was desired for the feasibility pilot study; adolescent Latina youth were sought because they represent the largest and fastest growing ethnic minority group in the United States. Females were targeted because they demonstrate the highest rate of text messaging and were expected to be interested in participating. Phase I involved development of YEMAS and Phase II involved piloting of the system with Latina adolescents. Girls were eligible to participate if they were attending one of the participating high schools and self-identified as Latina. We contacted 96 adolescents; of these, 24 returned written parental consent forms, completed assent processes, and enrolled in the study.

**Results:**

YEMAS was collaboratively developed and implemented. Feasibility was established with Latina adolescents (N=24), who responded to four surveys daily for two two-week periods (four weeks total). Each survey had between 12 and 17 questions, with responses including yes/no, Likert scale, and open-ended options. Retention and compliance rates were high, with nearly 18,000 texts provided by the girls over the course of the pilot period.

**Conclusions:**

Pilot results support the feasibility and value of YEMAS, an automated SMS-based text messaging data collection system positioned within a secure university environment. This approach capitalizes on immediate data transfer protocols and enables the documentation of participants’ thoughts, feelings, and behaviors in real time. Data are collected using mobile devices that are familiar to participants and nearly ubiquitous in developed countries.

## Introduction

### Adolescents and Texting

Adolescents text more than they talk. No one communicates by text messaging more than adolescent females 12-17 years old, who *average* 4050 texts per month [1]. Boys of the same age average 2539, and to give context to this magnitude, the next highest texting average is 1630 texts per month among 18-24 year olds [1]. Among researchers, text messaging is growing in popularity as a strategy for collecting ecological momentary assessment (EMA) data. EMA are data reported in real time such that the respondent indicates in the moment of contact what they are feeling, thinking, or doing (eg, mood, alcohol use). EMA approaches also allow for collection of data in respondents’ natural settings [2,3]. EMA data help overcome challenges associated with recall-dependent data collection methods (ie, survey respondents reporting how they have felt the past two weeks, months, or years). Reducing recall bias by collecting data on emotions and events as they happen, EMA provides information and insights into the real life, in vivo experience of adolescents [4]. Using EMA allows an insider view on the range of emotions, thoughts, and activities that adolescents engage in throughout a single day. The challenges associated with recall-dependent data collection methods are well known, yet few valid and reliable alternative approaches exist. Until recently, those that have existed (eg, direct observation, hand-written diaries) have been costly, time consuming, susceptible to deceptive reporting (ie. falsified dates of when diaries were completed), and often unsustainable beyond the initial grant-funded project [2-6].

### EMA Data Collection

With technological advances, researchers have collected EMA data using electronic diaries completed with hand-held computers (ie, Palm Pilots), and more recently, cell phones. Interestingly, most researchers have used cell phones to collect and store momentary data, but have relied on manual, in-person uploading of data from the phone rather than taking advantage of the capacity of cell phones for real time data transfer, or wireless uploading that occurs at scheduled times during the study. For example, Shrier et al [5] collected momentary assessments of sexual health behaviors of adolescents using cell phones that were locked and programmed to probe the adolescent randomly for a week. The data were stored and later uploaded to a computer when the phone was returned to researchers. More recently, cell phones were used to capture real time data from youth about moods and physical activity with responses stored until the phone was returned [6].

Some researchers have reported using cell phones and real time text messaging technology to collect and transmit EMAs about adolescents’ alcohol use (N=26 [7]; N=20 [8]), children’s bleeding episodes (N=104) [9], overweight children’s self-management behaviors (N=141) [10], adolescent health information needs (N=60) [11], young adults’ drinking patterns (N=44 [12]; N=70 [13]), undergraduates irritable bowel symptom management (N=43) [14], and adults’ smoking cessation behaviors (N=31) [15]. However, published reports to date lack details regarding the system structure and the technicalities involved in transmitting EMA data. Other recently published reports describe the use of text messaging as a method for disease prevention intervention in developing countries [16], immunization reminders [17,18], asthma symptom management [19], prenatal care [20], and weight management [21]. These studies, while demonstrating the popularity of the use of text messaging in heath promotion, prevention, and intervention research, do not focus on the novel use of text messaging as a method of EMA data collection.

To augment the small but growing literature on the use of texting as a data collection tool, our team undertook a study to pilot and determine the feasibility of using an automated system for collecting EMA data from adolescents. We evaluated mood and related constructs four times a day for two weeks at a time. To accomplish this, a custom system had to be built within the university setting to securely and automatically send unique surveys to participants. Our study is the first, to our knowledge, to custom build a short messaging service (SMS; also known as texting assessment delivery system) for use with adolescents. This system was created to collect and transfer real time data about individual- and social-level factors that influence mental well-being. Benefits of this system include ease of data exchange, automation to reduce errors related to human effort, contextualized understanding of health behaviors, and the ability to capture data across numerous data points for longitudinal and trajectory data analysis.

In this article we describe the collaborative development of Youth Ecological Momentary Assessment System (YEMAS), and the specific features, including linkage to a cloud communication service provider, that foster efficient data collection, management, and storage. We present feasibility and compliance data from our pilot study of 24 Latina adolescents, and conclude with suggestions regarding the ways in which this system can be used in behavioral health research with adolescents.

## Methods

### Latina Adolescent Females in this Study

This was a two-phase study that included development of YEMAS and a feasibility pilot with Latina adolescent females. Relative homogeneity of participants was wanted for the feasibility pilot study. Adolescent Latina youth were sought because they represent the largest and fastest growing ethnic minority group in the United States [22]. Females were targeted because they demonstrate the highest rate of texting [1] and were expected to be interested in participating. In fact, findings from a 2012 study suggest that SMS and social media are pervasive among Latino youth and that the youth view text messaging as a credible and essential method of communication [23].

### Phase I–Development of YEMAS

Our multidisciplinary team was comprised of academic faculty representing the fields of nursing, public health, and psychology. Also on the team were information technology (IT) professionals and graduate students. The research team identified features of the system that would yield useful data, and the IT team members were able to advise the research team on which features could be accomplished given the realities of technological capabilities. There were two options for the system that were explored: (1) development of all aspects of the system within the university structure, and (2) involvement of a nonacademic third party (ie, telecommunication system) to support the secure transfer of texted data. The pros and cons of both scenarios were carefully considered in the context of security concerns, priorities for protecting research study participants, and the data they share via text. Importantly, the nonacademic third party Twilio, a cloud communication service provider that facilitates both the text messaging and data management/exporting, was able to document texting patterns, but their staff was not able to access actual text content. This was important to ensure that only the research staff at the university had access to the raw, texted data, rather than staff at the telecommunications company. Creating a full SMS delivery system requires tremendous financial investment and resources including purchasing the hardware, software systems, and phone numbers in addition to the time intensive development of the application. By utilizing a cloud communication service such as Twilio, a system like YEMAS can offer a cost-effective way to achieve the primary goal of delivering and receiving content via SMS. YEMAS was developed to leverage this cost-effective strategy. Also, with a university-based system, we were able to capitalize on internal security and server features, establish the Web-based assessment management system on a university Web address, and use secure university log-in systems to control access.

Via eight in-person meetings and regular electronic communication, the research team identified key system features and worked through challenges that were encountered. For example, an early idea was to use the email infrastructure to deliver and receive text messages; however, it became clear that the scope of data being collected, along with limited capacity of data management and organization via email, necessitated an alternative approach. Our initial meeting identified what the researcher was envisioning, and provided the programmer with information from which to research possible solutions. Follow-up meetings presented the possible solutions, with their respective pros and cons. Discussions were open with everyone on the team expressing opinions and preferences, using previous literature and the state of technology as guides. Thus, YEMAS was developed as a Web-based survey application that facilitates the sending and receiving of SMS texts by integrating with a Web-service application programming interface (API) solution, Twilio. Upon implementation, the research team communicated regularly so that the programmer became quickly aware of any challenges being encountered by the implementation team members during the pilot study.

YEMAS is a custom built SMS-based EMA delivery system comprised of four components (see Textbox 1). Technically, YEMAS operates in a Windows 2008 Server environment utilizing Oracle as the database and ColdFusion as the programming language (contact the authors for additional technical inquiries).

**YEMAS components.**

- *Questions–*This is where all survey questions and corresponding response options are entered and stored.
- *Surveys*–This section houses all of the surveys created from the question bank. Each survey can be configured with the following parameters–
  - Delivery type (daily or weekly)
  - Delivery time schedule (in 5 minute increments)
  - Question order (random or predetermined)
- *Messages–*This section has three parts**–**
  - *SMS Queue,* where surveys are sent once they have been assigned a day and time for release and have been made active.
  - *SMS Results,* which captures each of the individual survey responses, from whom each response came, and the time each response was sent.
  - *SMS Log,* which displays the real time delivery log.
- *Participants–*The researcher enters the name/ID, email address, and phone number of each survey participant in this section. Participants are assigned to surveys so that the research team can select which surveys specific study participants will receive.
- *Admin–*Once a survey is completed, the researcher uses this section to export the data into an Excel spreadsheet.

### How YEMAS Works

**Figure 1:**
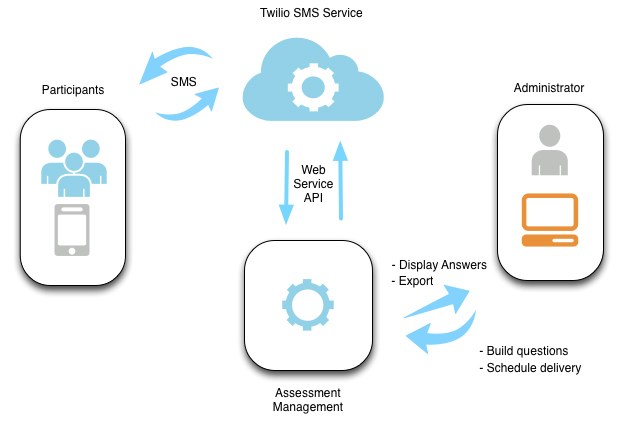
YEMAS SMS-based ecological momentary assessment data collection process.

The functional purpose of YEMAS is to send study questions to participants in an automated, scheduled manner and to enable participants to post SMS text messages back to the system in real time (Figure 1 shows the YEMAS data collection process). The process is as follows**–**The administrator (study Principle Investigator or research team member) constructs a survey with a list of questions and response options that are in a SMS friendly format and then schedules the survey for a specific date and time to be delivered. Each survey is assigned to a “rented” SMS number obtained from the Twilio Service (See Textbox 2 for an example survey, and Table 1 for an example survey schedule. Multimedia Appendix A shows example EMA questions, response options, and delivery frequencies.). The cost of a SMS number (ie, phone number from which the texted questions are sent) from Twilio was $1 per month.

Once survey questions are scheduled in the system by the researcher, the YEMAS completes an automated server-side schedule task that builds a daily delivery queue. When the survey is due to be delivered, the system sends each question as a SMS text message to the participant’s phone number via the Twilio SMS API. The API is a set of programming standards for accessing a Web-based software application or Web service. It is a software-to-software interface, not a user interface. With APIs, apps talk to each other without any user knowledge or intervention. In this way, the YEMAS interacts with Twilio to effectively send and receive text messages with numerous study participants simultaneously.

When the participant sends an answer as a SMS text message, Twilio takes the SMS text message and posts it via hypertext transfer protocol to the Coldfusion-powered SMS end point in YEMAS. The system then looks at the form data to confirm that it can determine the participant’s phone number (identification number) and the assigned SMS number (which is unique to the survey delivered at a specific time of day and day of the week). Once both numbers are confirmed, the returned answer (either the number corresponding with the appropriate response option, the word/phrase corresponding to the appropriate response option, a combination of the number and word/phrase, or an open-ended response) is stored in the database. The collected data can be viewed to observe actual responses, response rates, and trends, and can then be exported to a spreadsheet file for further analysis.

Importantly, the subsequent question in the survey will not be sent by the system until the participant provides a text response to the initial question. In this way, a set of questions is not sent all together; rather, questions are sent sequentially following responses that are received (note–this is similar to what many private sector businesses currently use to evaluate their services by text; a question is sent and when a response is provided, the next question is delivered). Therefore, if a participant chooses not to respond to a question, that survey (for that time of day only) is stopped. Investigators can provide the response option, “skip this question,” to allow participants to move on to the next question. To avoid receiving responses for survey questions sent the previous day, each survey had a unique phone number from which the survey was delivered and this number changed at midnight, in addition, each text was time and date stamped to allow for monitoring responsiveness.

**Example survey.**

- How HAPPY were u feeling just before u got this txt?

1.Not at all, 2.A little, 3.Quite a bit, 4.Extremely

- How TIRED were u feeling just before u got this txt?

1.Not at all, 2.A little, 3.Quite a bit, 4.Extremely

- How ENERGETIC were u feeling just before u got this txt?

1.Not at all, 2.A little, 3.Quite a bit, 4.Extremely

- WHERE were u just before u got this txt?

Open-ended

- How SAFE do u feel where u r right now?

1. Unsafe, 2. Somewhat safe, 3. Very safe

- Were u ALONE just before u got this txt?

1. Yes, 2. No

- Were u with ur MOM or DAD just before u got this txt?

1. Mom, 2. Dad, 3. Both, 4. Other adult 5. No one

- Describe something stressful u had to cope with since the last txt survey.

Open-ended

- Would u say u r generally healthy 2day?

1. Yes, 2. No

- How many hours of sleep did u get last night?

Open-ended

- Do ur parent(s) know who most of ur friends R?

1. Yes, 2. No

**Table 1:** Example survey schedule.

Weekdays (Monday-Friday)	Weekends (Saturday-Sunday)
6:30 AM	10:30 AM
2:30 PM	3:00 PM
6:00 PM	6:00 PM
9:00 PM	9:30 PM

### Pretest of YEMAS

We pretested YEMAS three times with three teens (not involved in the pilot study) and six research team members. The first pretest with teens consisted of a 12-question survey sent twice in one day (morning and evening), which allowed us to observe how the texting would work and qualitatively assess perceived response burden. At the end of this pretest, the three teens were asked four questions, also by text, to assess their experience. A second pretest with team members allowed us to refine question wording. We also ensured that all survey questions fit into a single SMS text message (met character number restrictions), and that the introductory text for each survey (ie, “Good afternoon. It’s time for a survey”) was sent before the first survey question. A final pretest with teens focused on ensuring that survey times were appropriate, survey questions were sent and received at the programmed time, and that once a response was received, the subsequent question followed immediately.

### Phase II–YEMAS Feasibility Pilot Study

A feasibility study was conducted to establish the utility of YEMAS and to test data collection and management over an extended period of time with adolescent participants.

### Setting

Recruitment of study participants took place in two urban public high schools in St. Paul, Minnesota. Both schools were similar with respect to the number of Latino students, but one had greater representation of students of color, and students qualifying for free or reduced lunch.

### Participants and Recruitment

Girls were eligible to participate if they were attending one of the participating high schools and self-identified as Latina. School records indicated that there were 100 eligible girls across the schools; of these, 96 were contacted by a research team member about the study and invited to participate. There were four girls that did not have confirmed contact, because they were possibly not at school on the days recruitment occurred, and they did not respond to the mailed information about the study. A research team member actively recruited during school lunch periods and at outreach events for Latino parents. There were 27 adolescents that indicated interest; of these, 24 returned written parental consent forms, completed assent processes, and enrolled in the study. Both schools and the University Institutional Review Board provided approval for this study. Participant identifiable information was kept separate from the raw EMA texted data, which were linked to the telephone number from which the texting originated.

### Measures and Pre- and Postsurvey Items

Upon enrollment, participants completed a baseline survey including demographics and self-reported health behaviors, mood, perceived stress, coping, social networks, and family and school connectedness. Standardized measures with established reliability and validity with adolescents were used in the survey. The survey consisted of 241 questions ranging from assessment of perceived ability to cope, to connections with family. A postsurvey was administered at the end of the study. A majority of participants completed this survey by computer via REDCap, a secure, Web-based application for building and managing computer surveys and databases [11]. The survey required approximately 40 minutes to complete. Through REDCap, each participant received an individual link sent directly to her email account. Once they clicked the link they could start the survey and stop and come back to it at any time. A minority of participants preferred to complete the survey manually, with their responses subsequently entered by the research assistant.

### Procedures

Enrolled girls were given detailed instructions regarding the texting process; those without their own cell phone (n=4) were provided a phone limited to texting capabilities and were instructed regarding its use. A mini-manual was provided to each girl, describing the study, the texting process, example texting items, and how to contact the research team with questions. There were two texting protocols (A and B) that were used to examine different strategies that could optimize response rates. Girls were randomly assigned to start with Protocol A or Protocol B, which they completed for two weeks, and following a two-week break, they crossed-over to the other protocol. Protocol A required the girls to respond as quickly as possible to the texted questions, grouped and sent four times per day (ie, signal sampling). Protocol B advised the girls to provide open-ended texts to describe how they were feeling when particularly positive or negative things were happening in their lives (ie, event sampling). In addition, Protocol B included the four scheduled surveys of Protocol A, but girls were informed they could respond to the texts whenever they wanted. Each morning they received a text reminding them to offer open-ended texts that could be in response to the first scheduled survey that day.

The survey measures delivered via SMS text messages included Likert scales and open-ended questions assessing individual- and social-level factors influencing well-being (eg, affect, social context, parental monitoring). Some of the measures were derived from established longitudinal surveys of adolescents (ie, AddHealth, Minnesota Student Survey), and others were developed explicitly for this study. All questions were adapted for texting purposes, using common abbreviations for specific words (for example, the word “you” shortened to “u”).

How often a survey item was delivered was based on the kind of behavior and/or event being assessed and the likelihood of occurrence throughout the day and week for adolescents. For instance, items assessing momentary mood and activity participation, interest, and motivation were assigned a maximum frequency of two times per day. Alternatively, items assessing parental knowledge of activities and bullying were assigned frequencies ranging from once per week to three times per week. An entire survey instrument (ie, Positive and Negative Affect Schedule feelings scale) was not completed in one survey, but all items were asked via EMA, spread across different times of day and days of week. The response burden was considered (ie, open-ended items that required more effort were not assessed more than once per day), the number of questions for each survey ranged from 11 to 21, with each survey designed so that all questions could be answered in five minutes or less (the Multimedia Appendix A shows the complete list of EMA items and frequency with which they were administered). It is important to note that all participants received identical surveys for the same time period (ie, Monday morning, Friday afternoon) to facilitate comparison of EMA data across participants.

Participants were informed of the date that the SMS text surveys would begin. Additionally, a study research team member provided personalized reminder texts or calls, based on the participant’s preference, to increase response rates and study involvement. Participants were provided with reminders before each protocol resumed after the two-week break and once a week throughout each two-week study period. Text responses were monitored every other day, and if a participant had not responded to texts for up to 2 days, they were contacted to confirm receipt of texts and to determine that there was not a problem with their phone. This monitoring, completed by the research assistant, was not required from a system perspective, but contributed to obtaining high quality pilot study data. On a few occasions a girl’s phone number had changed. When the system was updated with the correct number their texting responses resumed.

To pilot test the YEMAS, a research team member received all the texts that were sent, in this way system issues were readily identified and addressed. For example, evening surveys were to occur no later than 9:30 pm, but on one occasion the survey was texted around 11:00 pm. On another occasion, the same question was sent multiple times only seconds apart. These problems were identified and corrected with careful monitoring through the study. All problems were not identified during pretesting of the system primarily because selected surveys, but not all 28, were pretested. In addition, telecommunications complications may be specific to phone models or companies, and these may not arise during a pretest.

### Compensation Structure

Participants received modest compensation ($5 gift card) for completion of the REDCap surveys. To encourage texting responses, participants were provided entries into a drawing for an iPod touch, the number of entries per person correlated with their percentage of text responses to reward responses.

## Results

### Participant Demographics

Participants were between 14 and 17 years old (mean age 15). In this feasibility study, four participants required a cell phone to be provided for survey completion. Among those girls who had a phone, all had unlimited texting plans and did not require financial support to upgrade their cell phone service plan. All but one participant had Internet access at home or a friend’s house, which was necessary for the completion of the pre- and postsurveys. The Internet was available at both schools and one participant utilized a school computer lab to complete her surveys.

### Feasibility

In order to establish the feasibility of YEMAS, we conducted analyses addressing protocol adherence, examining the number of responses to each protocol, the percentage of participants who responded to texts daily, and the percentage of questions completed during a given two-week data collection period.

The number and percentage of participants who responded to texts daily is provided in Table 2 for Protocol A (signal-based sampling) and Protocol B (event-based sampling) for three “rounds” of texting. Girls were recruited over time, and for this reason they entered the study during Round 1 or Round 2. All girls who participated during Round 1 were new recruits who were randomly assigned to receive Protocol A or Protocol B. Girls who participated during Round 2 were a mixture of new recruits and girls who were completing the second protocol (ie, a cross-over design). Girls who participated in Round 3 were all completing their second protocol.

As shown in Table 2, participants completing Protocol A responded to surveys on a greater proportion of days. This adherence to Protocol A occurred regardless of whether participants had been assigned to the sequence of Protocol A followed by Protocol B or vice versa. However, the first group of recruited girls (Round 1) had high proportions of responses regardless of assigned protocol. This could reflect greater interest in the study among girls recruited early as compared with girls recruited in future rounds, or it could be a function of response burden (Round 2 represents new recruits and the cross-over of Round 1 participants, whose interest in the study might have waned over time).

**Figure 2:**
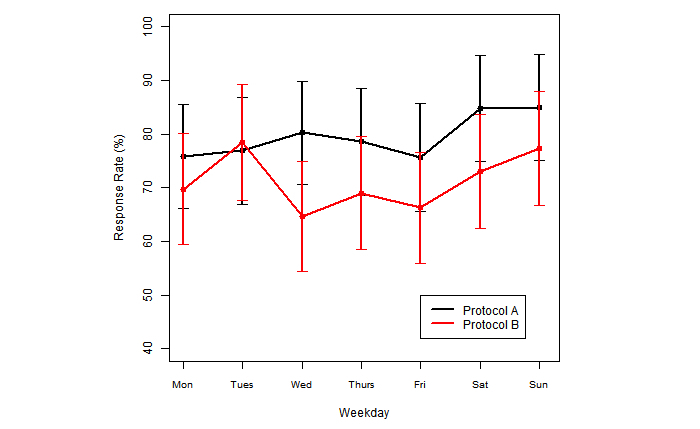
Weekday average response and 95% CI.

Table 3 provides the percentage of questions completed within each protocol. It is interesting to note that the rate for Table 3 is based on how many questions are responded to at each time point, as compared with the rate for data in Table 2 being based on how many days texts were responded to, thereby offering insight into responses across and within days of texting data collection. The data reveal that Protocol A (signal-based sampling) yielded a slightly higher response rate among adolescents (see Table 3). Figure 2 shows a weekday average response. It is possible that Protocol B (event-based sampling) resulted in greater burden for participants. This is consistent with findings from other studies that have shown greater response rates for signal-based sampling than event-based sampling, even for events that appear well defined, such as asthma attacks [2].

**Table 2:** Texts and mean percentage of daily responses among participants by protocol and data collection period.^a^

	Protocol A Signal-based sampling		Protocol B Event-based sampling	
	Participants, n (texts, n)	Mean text response rate, % (range, %)	Participants, n (texts, n)	Mean text response rate, % (range, %)
Round 1	7 (4963)	99 (94-100)	7 (2009)	96 (94-100)
Round 2	11 (6328)	84 (47-100)	8 (2746)	11 (11-100)
Round 3	2 (323)	53 (40-100)	5 (1925)	56 (13-87)

^a^Only participants who responded to at least one survey occasion during the round of surveys were included in these numbers.

**Table 3:** Mean percentage of questions completed by protocol at each time point.

Protocol	Response rate	95% CI
A (signal-based sampling)	79.59	70.53-88.66
B (event-based sampling)	71.21	61.97-80.45

## Discussion

### Purpose of the Study

The purpose of this study was two-fold: (1) to develop a Web-based automated system for real time SMS data collection, and (2) to pilot this system with a subset of Latina adolescents. This preliminary study provided important information, resulting in recommendations for next steps utilizing text surveys for collecting real time longitudinal data.

Our research team developed and pilot tested the YEMAS. This project has moved the field of EMA data collection forward. Previous studies focused on collecting and storing EMA data via Palm Pilots and cell phones [2,5], however, few researchers have reported use of an infrastructure that allows for real time assessments [6,7]. The findings of this study suggest that it is possible to employ immediate data transfer protocols, taking advantage of capabilities of cell phone and texting technology, rather than using outdated methods of storing data that require manual or scheduled remote uploading at various points during the study. Achieving immediate awareness of responses, as well as monitoring compliance daily, allowed timely follow-up with participants.

This study also has implications for the ways in which data are collected from adolescent participants in descriptive and intervention studies. In particular, studies promoting the emotional well-being of adolescents may uniquely benefit from capturing real time, in vivo momentary assessments via SMS text messaging, which promote a more accurate picture of the frequent fluctuations in affect experienced by many adolescents. SMS text messaging data could be used to compare against and augment traditional forms of data collection, such as periodic reports of mood during clinic visits or annual nationally representative cross-sectional surveys of youth. Data triangulation could offer important, new insights informing health behavior promotion and intervention research. In developing and piloting YEMAS, our project team learned many valuable lessons specific to the logistics of ensuring smooth system functioning.

As a population who frequently uses cell phones, many of us have experienced the problems and frustrations associated with sending text messages. Unfortunately, some of these issues will also arise when using SMS text messages and cell phones as a platform for collecting data (eg, EMA data). Even with programming accuracy, the nature of cloud-based SMS telecommunication allows for occasional mishaps, such as text responses arriving out of order. The type of automated system for data collection used in this study proved to be a very useful and important tool that will undoubtedly become more popular for collecting real time information from study participants. This type of system reduces the costs (ie, approximately US $.01/text) and offers capacity for large-scale automated EMA data collection.

### Participants’ Text Responses

An intriguing aspect of using SMS text messages to collect survey data was that, unlike many computer-based or paper-based surveys, participants’ responses were not limited to a single number or rating. Participants frequently provided contextual details to elaborate on their responses. While this type of information will be useful as we create future studies, it also created some difficulty with the initial data analysis, requiring closer inspection of many of the responses because automatic coding proved to be inaccurate (eg, instead of simply texting “yes,” the respondent said, “yes, I am just hanging out,” which required manual recoding as “yes”). The result was a time-consuming data cleaning process that reduced the 21,000 texted responses to 17,717 in the final cleaned data set. Some common reasons for removing a texted response were duplicates (text of a “y” sent followed by “yes” to the same question), split texts (one answer split across two texts).

The research team had to be familiar with texting shorthand linguistics, which required the occasional input from people outside of the study to determine what some responses meant. Most people are familiar with the term “lol” (“laughing out loud”), but others such as “Idk” (“I don’t know”) or symbols such as <x_x> (“smiling”) required investigation.

One final, and less frequent, problem was responses that appeared to be messages intended for other recipients. This problem is unique to this type of study because we are using text messages sent on participants’ cell phones, which are also being used for any number of other activities (ie, texting friends, talking on the phone, listening to music, taking pictures, or going on the Internet). Given the nature of text messaging and the use of texts as the preferred way for adolescent communication, it is probable that participants received survey questions at the same time they received or were sending messages to their friends or family. Sending a message to the wrong person is possible, especially when someone is interrupted in the midst of writing that message. Therefore, it is likely that some of the responses received were intended for other people (for example, the text message “ok,” which is commonly used in everyday life, did not make sense in response to a “yes” or “no” question, such as “Did a teen threaten u at school 2day?”). This is a potential problem for any study utilizing communication technology; however, in our study these occurrences were rare, and comprising less than 0.5% of the 14,000 responses. In addition, these errors were easily identified during the cleaning process. Future studies could include algorithms prompting automated follow-up when nonsensical or incongruent responses occur.

This pilot study thus yielded many valuable insights. Our study advanced understanding that adolescents respond with higher rates to signal-triggered data collection rather than event-based sampling. Additionally, the development of a university-based system linked to an existing Web-based API (ie, Twilio) proved to be efficient. This pilot study led to discussions with Twilio about customization and potential improvements to their system that could strengthen YEMAS in future studies. Finally, a key success from this pilot study was the creation of the infrastructure for a system that can be used to simultaneously survey numerous participants using SMS, a technology that is very accessible and already in widespread use across all socioeconomic levels nationally and globally.

### Uses for YEMAS in Health Research

There are numerous uses for the YEMAS as a research tool in health research. From descriptive studies exploring new phenomena to randomized controlled intervention trials, EMA data collected via text messaging have potential to provide both new and complementary insights. As an addition to traditional self-report pre- and postintervention survey data, EMA data offer real time insights that can be efficiently collected and analyzed within and across multiple time points. Prospectively, these data could be useful in describing day-to-day fluctuations in characteristics or variables that are difficult to capture effectively in traditional survey approaches, including moods, feelings, or fluctuations in social relationships and interactions. Indeed, momentary sampling is more appropriate for factors that do not remain constant over time. Therefore, a system like YEMAS is essential for collecting data on these factors, independently, or as a complement to data collected through traditional methods.

Although YEMAS was designed initially for use with adolescents, the structure and system that have been created could be used with any research population. The survey questions and frequency of their delivery can be determined by the research team and guided by specific study aims. Text messaging is a common communication tool in the 21st century among all ages and ethnicities [23,24]. As a Web-based tool, the YEMAS can facilitate data collection from any target study population anywhere in the world. The availability of apps that facilitate texting at no cost (ie, Blackberry Messenger, What’s App) is contributing to the ways in which SMS technology can be combined with Web-based systems for effective contextualized data collection in behavioral health research.

### Limitations

The study has limitations to consider when interpreting results. The small sample size was appropriate for a pilot study, but limits generalizability of the findings. Similarly, the homogeneity of the adolescents (Latina females) is a strength, but limits relevance to non-Latina adolescents and males. Finally, items were asked within each EMA survey in a sequential manner, rather than randomized in order. This is a limitation that could be avoided with additional sophisticated programming that would allow for within survey selection of sequential items (ie, those that are in follow-up to a previous question) and randomization of the order of remaining questions. This study was focused on feasibility and therefore provides an important foundation upon which future research can build and establish EMA data collection best practices among adolescents.

### Conclusions

This study demonstrates the feasibility of conducting EMA through SMS (texting) among adolescents. This approach capitalizes on immediate data transfer protocols and enables the documentation of participants’ thoughts, feelings, and behaviors in real time. Data are collected using mobile devices that are familiar to participants and nearly ubiquitous in developed countries. Further, our research team has developed YEMAS, a content management system that facilitates efficient and automated data collection, management, and storage. The YEMAS approach to EMA is broadly applicable to studying the health behavior of individuals who use texting technology. The tools and protocols described in this manuscript thus have the potential to complement or transform existing approaches to studying self-reported phenomena among individuals that may fluctuate over time.

## Multimedia Appendix 1

Texted items by days and times administered.

## Abbreviations

API: application programming interface
EMA: ecological momentary assessment
IT: information technology
SMS: short messaging service
YEMAS: Youth Ecological Momentary Assessment System
