# Supplementary material for: Teenagers and Texting: Use of a Youth Ecological Momentary Assessment System in Trajectory Health Research With Latina Adolescents
Source: JMIR Mhealth Uhealth. 2014 Jan 24;2(1):e3. doi: 10.2196/mhealth.2576 (PMC4114422; doi:10.2196/mhealth.2576)
Supplement: Supplementary file 1 [file mhealth_v2i1e2_app1.pdf]

Item Wording	Days Administered							Times Administered				
	M	T	W	Th	F	S	S	AM	AN	EVE	PM	AS
How HAPPY were you feeling just before u got this txt?	X	X	X	X	X	X	X	X	X	X	X	X
How EXCITED were you feeling just before u got this txt?	X	X	X	X	X	X	X		X		X	X
How STRESSED were you feeling just before u got this txt?	X	X	X	X	X	X	X	X	X	X	X	X
How MAD or ANGRY were you feeling just before u got this txt?	X		X	X	X	X	X		X	X	X	X
How NERVOUS or ANXIOUS were you feeling just before u got this txt?	X		X	X	X	X	X		X	X	X	X
How SAD were you feeling just before u got this txt?	X			X	X	X	X		X	X	X	X
How RELAXED were you feeling just before u got this txt?	X	X	X	X	X	X	X	X	X		X	X
How TERRIFIED were you feeling just before u got this txt?	X	X	X		X	X	X		X	X	X	X
How TIRED were you feeling just before u got this txt?	X	X	X		X	X	X		X	X	X	X
How ENERGETIC were you feeling just before u got this txt?						X	X	X				
How INTERESTED were you feeling just before u got this txt f?						X	X	X				
How DISTRESSED were you feeling just before u got this txt?				X			X	X				
What were you DOING right before u got this txt? (Choose your main activity)	X	X	X	X	X	X	X		X	X	X	X
How LONG have you been doing this activity?			X		X	X			X	X	X	X
How MUCH FUN is this activity?	X	X			X		X		X		X	
Are you doing this activity because you want to do it?		X				X				X	X	X
Are you doing this activity because your FRIENDS want you to do it?		X	X	X	X	X	X		X	X	X	X
Are you doing this activity because your PARENTS want you to do it?	X	X	X	X	X	X	X		X		X	X
Are you doing this activity because your TEACHER wants you to do it?	X	X		X	X				X		X	X
WHERE were you just before u got this txt?												
How SAFE do you feel where you are right now?		X										X
How FAR are you from your home right now?					X		X	X	X	X		
